# Differential Coordination Chemistry with Hydrogen Peroxide: A Pathway Toward Selective Separation of Tungsten from Molybdenum

**DOI:** 10.3390/ma19091822

**Published:** 2026-04-29

**Authors:** Yiying Wu, Yuqing Qiu, Jigang He, Xingyu Chen, Ailiang Chen, Xuheng Liu, Jiangtao Li, Wenjuan Zhang, Fenglong Sun, Zhongwei Zhao

**Affiliations:** 1 School of Metallurgy and Environment, Central South University, Changsha 410083, China; 2 Key Laboratory of Hunan Province for Metallurgy and Material Processing of Rare Metals, Changsha 410083, China

**Keywords:** tungsten, molybdenum, hydrogen peroxide, selective leaching, differential coordination

## Abstract

The efficient separation of tungsten and molybdenum represents a pivotal challenge for the effective, high-value-added utilization and recycling of these strategic metal resources. Developing clean and recyclable separation processes has become a major focus of research, reflecting a growing emphasis on sustainability. This study proposes a method for the efficient and deep separation of molybdenum and tungsten from tungstate-based mixed oxides by leveraging their differential coordination properties with hydrogen peroxide. The composites prepared by mechanical mixing were characterized using techniques such as ICP, SEM, XRD, and Raman spectroscopy. The results demonstrated a significant difference in the dissolution behavior of MoO_3_ and WO_3_ in hydrogen peroxide, indicating a substantial coordination disparity between MoO_3_ and WO_3_ toward H_2_O_2_, which can be effectively exploited for Mo/W separation. Additionally, hydrothermal synthesis was employed to simulate the separation under more realistic conditions. In this study, hydrogen peroxide was selected as an effective reagent for separation, and the influence of multiple variables was systematically evaluated. The results demonstrated that under optimal conditions—specifically at a molar ratio nMo/nW = 40, a temperature of 65 °C, nH_2_O_2_/nM = 1.25 and a reaction time of 1.5 h—a maximum separation factor of 124 between tungsten and molybdenum was achieved. This process exhibits significant potential for industrial application due to its low consumption of H_2_O_2_, large separation factor, and cost-effectiveness.

## 1. Introduction

Tungsten (W) and molybdenum (Mo), as strategically critical rare materials, are extensively utilized in a wide spectrum of vital applications within the national economy and defense industry owing to their exceptional properties, including high melting points, superior hardness, excellent electrical and thermal conductivity, and unique chemical characteristics [1,2]. Tungsten serves as a critical material for cemented carbides, specialty steels, electrovacuum devices, and armor-piercing projectiles, while molybdenum is primarily employed as an alloying additive, catalyst, lubricant, and in applications such as thin-film transistors (TFTs) within the electronics industry. In nature, tungsten and molybdenum often co-occur through isomorphism in minerals such as scheelite and molybdenite. Owing to their similar atomic and ionic radii, as well as their nearly identical chemical properties, the separation of these two elements has long constituted a significant challenge in the field of hydrometallurgy on a global scale [3,4].

Although conventional tungsten–molybdenum separation technologies, such as sulfide precipitation, selective precipitation, ion exchange, and solvent extraction, have been employed in industrial applications, they all exhibit certain limitations [5,6,7,8]. As exemplified by the thiomolybdate method employed by Li et al. [9,10,11] the process has been widely adopted in Chinese industry due to its shorter process flow and high purification efficiency. However, the method requires a large consumption of sulfiding agents (with a Mo/S molar ratio of approximately 10–20), which not only causes environmental pollution issues–such as generating hazardous waste MoS_3_ and toxic hydrogen sulfide gas–but also incurs high costs for reagents and waste treatment. These limitations become particularly pronounced when the mass ratio of molybdenum to tungsten trioxide exceeds 2%. Therefore, these methods exhibit limited applicability for separating molybdenum from tungstate solutions with a high Mo/W mass ratio. Furthermore, some emerging tungsten–molybdenum separation technologies have been developed; foe example, Zhao et al. [12] proposed a novel W-based IIP adsorbent which had abundant recognition sites and imprinted cavities. Under the optimal adsorption conditions, a separation factor of 75 was achieved for a more efficient and environmentally friendly W-Mo separation process. However, W-IIP currently faces limited recycling ability, cost implications and potential scalability constraints.

Another method for separating molybdenum from tungsten is based on the differences in properties between peroxomolybdates and peroxotungstates. Hydrogen peroxide (H_2_O_2_) effectively suppresses the precipitation of tungstic and molybdic acids under acidic conditions and inhibits the formation of tungsten–molybdenum heteropoly acids. Furthermore, it reacts with tungsten or molybdenum to form the corresponding peroxometal acid complexes. Among these, peroxomolybdates can be preferentially extracted using neutral or alkaline extractants. As a cost-effective and environmentally benign complexing agent, the introduction of hydrogen peroxide does not introduce ions that are harmful to subsequent processes or the environment [13,14]. Compared with the sulfide method, this process has significant advantages in terms of environmental friendliness and economy, especially suitable for treating tungstate solutions with a high molybdenum content. However, this method still faces problems at present, such as a relatively high tungsten co-extraction rate, and methyl isobutyl ketone (MIBK) being prone to volatilization and having a large water solubility, resulting in severe loss of the organic phase. Therefore, this process has not yet achieved large-scale industrial application [15]. A growing number of studies have recently focused on the separation of W and Mo from peroxide-based complexation systems. A particularly striking phenomenon is that the high-valence oxides of tungsten and molybdenum (such as molybdenum oxide (MoO_3_) and tungsten oxide (WO_3_)) can form peroxometallic acid complexes in hydrogen peroxide solution—namely, peroxotungstic acid ([WO(O_2_)_2_]^2−^ or similar structures) and peroxomolybdic acid ([MoO(O_2_)_2_]^2−^) [15,16,17]. Xia et al. proposed a weak acid complexation method using a trialkyl phosphine oxide–tributyl phosphate (TRPO–TBP) mixed extractant system for the preparation of W(VI)–Mo(VI)–H_2_O_2_ solutions, focusing on the chemical behaviors of existing hydrogen peroxide complexation reactions and the aqueous species of W(VI) and Mo(VI) [11,15]. Zhang et al. developed evaporation deaminization and bipolar membrane electrodialysis methods to prepare W(VI)–Mo(VI)–H_2_O_2_ solutions from ammonium systems while preventing precipitation during acidification [14].

Zhang et al. [14] investigated a method for separating tungsten (W) and molybdenum (Mo) via thermal decomposition from a peroxide-containing acid solution. The thermal decomposition behaviors of pertungstic acid and permolybdic acid were studied separately, which confirmed that permolybdic acid exhibits significantly higher stability compared to pertungstic acid. V. P. Guro et al. found that in highly acidic solutions containing H_2_O_2_, the dissolution rates of molybdenum oxides (MoO_2_, MoO_3_) (0.6–0.7) remained 60 to 70 times higher than those of tungsten oxides (WO_2_, WO_3_). The dissolution rates of the tungsten oxides (0.01) were virtually negligible, indicating their extreme stability and resistance to H_2_O_2_-mediated dissolution under acidic conditions [17]. Although both can form peroxo complexes, in-depth studies have revealed that these complexes exhibit subtle yet critical differences in thermodynamic stability, kinetic rates of formation and decomposition, and spatial geometric configurations.

These differences provide us with a new perspective: the observed differences in the thermal stability of permolybdic acid and pertungstic acid, along with the distinct dissolution rates of MoO_3_ and WO_3_ in H_2_O_2_, have led us to hypothesize that variations may exist in the coordination of MoO_3_ and WO_3_ with hydrogen peroxide. This potential discrepancy suggests that modulating the dissolution conditions of these oxides in H_2_O_2_ may offer an effective strategy for achieving efficient tungsten–molybdenum separation. Therefore, it is essential to verify that the significant difference in the dissolution behavior of MoO_3_ and WO_3_ can effectively achieve tungsten–molybdenum separation. In this study, the forms of tungsten and molybdenum in industrial raw materials were simulated to evaluate the effectiveness of separation via H_2_O dissolution and to clarify the underlying mechanism. Finally, based on these findings, the separation conditions were optimized to identify the parameter set that yields the maximum separation factor.

In this study, we characterized both mechanically mixed pure MoO_3_ and WO_3_ and hydrothermally synthesized tungsten–molybdenum mixed oxides, which were subsequently applied for the separation of W and Mo, with simultaneous optimization of the separation conditions. The dissolution behavior was investigated using XRD, SEM, ICP, FTIR, Raman, and XPS, and the separation mechanism was thoroughly elucidated.

## 2. Experiment Section

### 2.1. Chemicals

This study utilized hydrogen peroxide (H_2_O_2_, 30% *w*/*w*, Sinopharm Chemical Reagent Co., Ltd., Shanghai, China) for separation and purification purposes. Sodium tungstate dihydrate (Na_2_WO_4_·2H_2_O, 99.5%, Macklin Biochemical Co., Ltd., Shanghai, China) and sodium molybdate dihydrate (Na_2_MoO_4_·2H_2_O, 99%, Sinopharm Chemical Reagent Co., Ltd.) were employed as the primary sources of tungsten and molybdenum, respectively. Molybdenum trioxide (MoO_3_, 99.95%) and tungsten trioxide (WO_3_, 99.95%) were purchased from Aladdin Holdings Group Co., Ltd. (Shanghai, China). Sulfuric acid (H_2_SO_4_, 95–98%, Chengdu Kelong Chemical Co., Ltd., Chengdu, China) was used in this study. All chemicals were of analytical grade and used without further purification.

### 2.2. Experimental Procedure

The solid and liquid phases obtained via the hydrothermal method were separated by filtration, and both the filtrate and the precipitate were collected for further analysis. The tungsten and molybdenum concentrations in the filtrate were quantified by inductively coupled plasma optical emission spectroscopy (ICP). The recovered precipitates were washed repeatedly with anhydrous ethanol and deionized water, then dried at 60 °C for several hours. The dried powders were gently ground in an agate mortar to obtain homogeneous samples, which were subsequently stored in a desiccator prior to Fourier-transform infrared spectroscopy (FT-IR), X-ray diffraction (XRD), and scanning electron microscopy (SEM).

Next, hydrogen peroxide was leveraged to achieve the selective separation of the W-Mo mixed oxide. For each experiment, 5.00 g of the mixed oxide was accurately weighed on an analytical balance, transferred into a 250 mL polypropylene vessel, and treated with a single addition of 10 mL H_2_O_2_. The slurry was then diluted to a final volume of 100 mL with deionized water. The mixtures were magnetically stirred at 500 rpm for a specified duration at a controlled temperature and then filtered to separate the filtrate and precipitate. The concentrations of tungsten and molybdenum in the filtrate were determined by ICP analysis, while the metal–oxygen bonding vibrations were characterized using Raman spectroscopy. The solid precipitates were washed several times with absolute ethanol and distilled water, and then dried at 60 °C for several hours. The dried powders were thoroughly ground in an agate mortar to obtain the final samples, which were stored for subsequent phase and morphological characterization by XRD, SEM, and XPS.

The distribution coefficients *λ* of tungsten and molybdenum were calculated according to Equation (1):

(1) $$\lambda =\frac{C_{0}\times V_{0}-C_{s}V_{s}}{C_{s}\times V_{s}}$$

*λ*—molar distribution coefficient; *C*_0_—initial concentration of metal in solution [M]; *C_s_*—final concentration of metal in solution [M]; *V*_0_—initial volume of the solution [mL]; and *V_s_*—final volume of the solution [mL].

The separation factor (*S_F_*) was also calculated as follows:

(2) $$S_{F}=\frac{\lambda_{W}}{\lambda_{Mo}}$$

*λ_w_*, *λ_Mo_*—molar distribution coefficient of W and Mo, respectively.

## 3. Results and Discussion

### 3.1. Dissolution and Separation Performance

**Figure 1:**
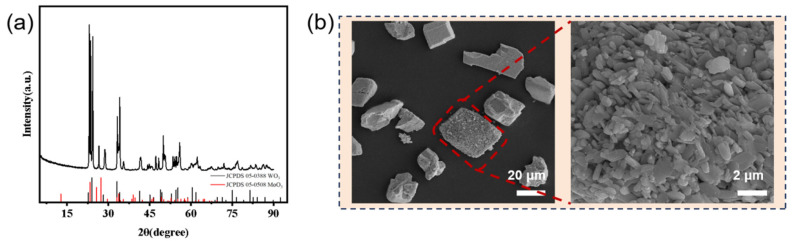
(**a**) XRD patterns of WO_3_, MoO_3_ and MoO_3_/WO_3_ composites; (**b**) SEM images of microstructure of MoO_3_/WO_3_ composites by mechanical mixing.

In this experiment, mechanical mixing was employed to prepare mixed oxide powders (n_H2O2_/n_M_ = 1:1), which were subsequently decomposed using hydrogen peroxide. The mechanically mixed powder comprising pure MoO_3_ and WO_3_ with a Mo/W mass ratio of 1 was subjected to XRD analysis to determine its phase composition (Figure 1a). Phase analysis confirmed the presence of both WO_3_ and MoO_3_, corresponding to standard reference cards PDF#05-0388 and PDF#05-0508, respectively. Characteristic diffraction peaks of WO_3_ were observed at 14.0°, 23.1°, 28.1°, and 36.8°, corresponding to the (100), (002), (200), and (202) crystal planes, respectively. Similarly, MoO_3_ exhibited reflections at 14.0°, 18.1°, 23.1°, 26.9°, 28.1°, and 36.8°, which can be assigned to the (002), (111), (020), (113), (202), and (222) planes, respectively. Therefore, the results demonstrated the successful synthesis of the materials. The SEM images in Figure 1b depict the morphological features of the MoO_3_/WO_3_ composite oxide synthesized via mechanical mixing. At low magnification (top), the composite exhibited a heterogeneous yet well-dispersed morphology consisting of irregularly shaped chunks (1–10 μm in size) and fragmented particles, with the red dashed box highlighting a representative composite agglomerate. The high-magnification inset (bottom), zoomed in on the marked region, reveals finer structural details: the WO_3_ matrix was uniformly coated with nanoscale MoO_3_ aggregates (50–200 nm in diameter), which appear as discrete, plate-like or granular particles adhering tightly to the WO_3_ surface. This structure—in which MoO_3_ nanoparticles were physically anchored onto the WO_3_ framework—reflects the mechanical mixing process’s ability to achieve intimate contact between the two oxides while preserving their individual morphological characteristics. The combination of macroscale (irregular chunks) and nanoscale (adhered particles) features aligns with the physical mixing mechanism, where mechanical shear forces facilitate the dispersion of MoO_3_ onto the WO_3_ surface without altering the intrinsic crystalline structures of either oxide—consistent with our prior XRD analysis.

**Figure 2:**
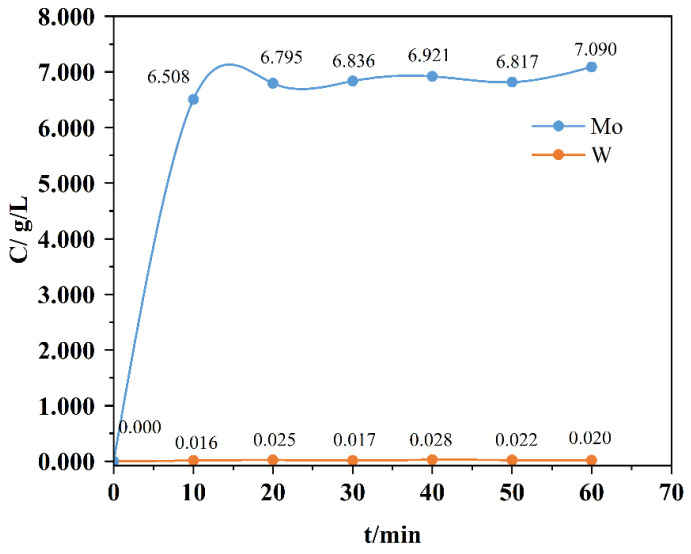
The effect of reaction time on the dissolution of pure tungsten oxide (WO_3_) and molybdenum oxide (MoO_3_) in H_2_O_2_.

Figure 2 illustrates the effect of reaction time on the dissolution of pure WO_3_ and MoO_3_ in hydrogen peroxide. As the reaction time increased, the dissolution rate of MoO_3_ rose continuously. Under the conditions of n_H2O2_/n_M_ = 1 and a dissolution temperature of 80 °C, the dissolution efficiency of MoO_3_ remained largely consistent as the time increased from 0.5 h to 2.5 h. Notably, a dissolution rate of 92% was achieved within just 0.5 h, and it stayed above 90% even after 2 h. However, a decline to 88% was observed at 2.5 h, possibly due to the reprecipitation of dissolved molybdenum species in other forms. In contrast, the dissolution rate of WO_3_ increased over time during the first 0.5 h to 1 h, reaching a maximum of only 0.5%. Beyond 1 h, the dissolution efficiency of WO_3_ gradually decreased, which may be attributed to the reprecipitation of dissolved tungsten compounds. The separation factor between MoO_3_ and WO_3_ in the mechanically mixed pure oxides using hydrogen peroxide reached 2463, demonstrating high selectivity under the applied conditions.

### 3.2. Investigation into Separation of Mixed Oxides and Mechanistic Insights

#### 3.2.1. Investigation into Separation of Mixed Oxides

**Figure 3:**
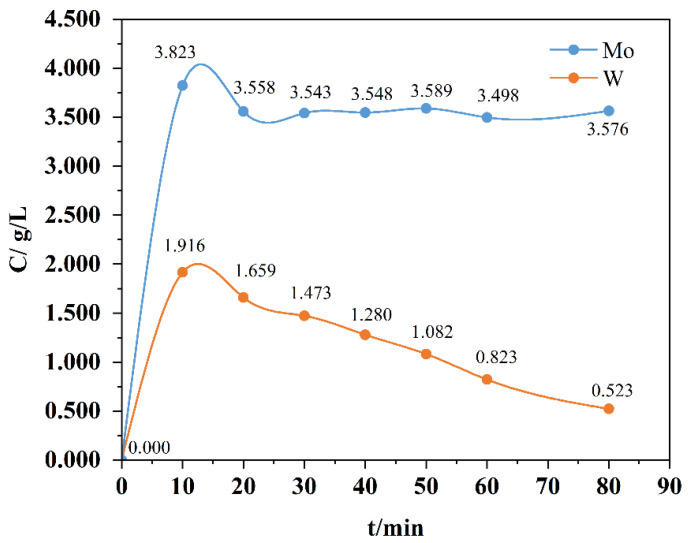
The oxide separated from H_2_O_2_ after hydrothermal treatment.

Based on the ideal experimental results obtained from pure reagents, it is preliminarily concluded that H_2_O_2_ exhibits significant potential for application in selective leaching. Under natural conditions, tungsten oxide and molybdenum oxide may not only be mechanically mixed. In order to further explore the separation effect under real-world conditions, we mixed tungsten oxide and molybdenum oxide via the hydrothermal method to simulate natural conditions (Figure 3). Accordingly, experiments were conducted under the conditions of n_H2O2_/n_M_ = 1 and a leaching temperature of 80 °C. The leaching efficiency of hydrothermal powders with n_Mo_/n_W_ = 1 in H_2_O_2_ was compared over time periods ranging from 0.5 to 2.5 h to evaluate the separation performance. As observed from the kinetic curves, the dissolution of molybdenum reached equilibrium within 20 min, while the dissolution rate of tungsten continued to decrease rapidly. Specifically, the dissolution rate of tungsten dropped from 27.45% to 7.49% within 60 min, with the decreasing trend persisting thereafter. This phenomenon may be attributed to the formation of a tungsten-enriched layer encapsulating molybdenum oxide particles in the mixed oxides, thereby inhibiting the further dissolution of molybdenum. Simultaneously, the dissolved tungsten may initially co-dissolve with molybdenum followed by gradual depolymerization and reprecipitation, leading to the continuous decline in the tungsten dissolution rate.

#### 3.2.2. Mechanistic Insights

**Figure 4:**
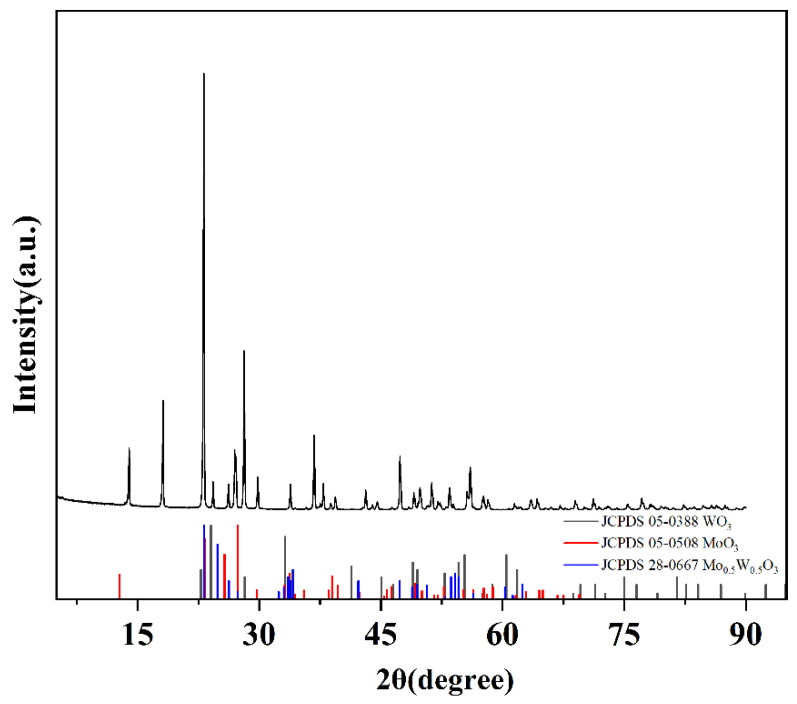
XRD pattern of hydrothermal powder synthesized from sodium molybdate and sodium tungstate at Mo/W molar ratio of 1.

To investigate the transformation induced by the reaction, oxide powder mixtures with a molar ratio of 1:1, WO_3_ and MoO_3_, were prepared and analyzed. Phase composition and morphological evolution were examined using XRD and SEM. Figure 4 shows the XRD pattern of the powder hydrothermally synthesized from sodium molybdate and sodium tungstate at a Mo/W mass ratio of 1. Phase analysis confirmed the presence of WO_3_, MoO_3_, and W_0.5_Mo_0.5_O_3_, which correspond to the standard reference cards PDF#05-0388, PDF#05-0508, and PDF#28-0667, respectively. The XRD pattern exhibited diffraction peaks of WO_3_ at 14.0°, 23.1°, 28.1°, and 36.8°, corresponding to the (100), (002), (200), and (202) crystal planes, respectively. Peaks attributed to MoO_3_ were observed at 14.0°, 18.1°, 23.1°, 26.9°, 28.1°, and 36.8°, which can be indexed to the (002), (111), (020), (113), (202), and (222) planes. Additionally, the characteristic peaks of W_0.5_Mo_0.5_O_3_ were detected at 23.1°, 26.2°, and 33.8°, assigned to the (110), (111), and (112) planes, respectively.

**Figure 5:**
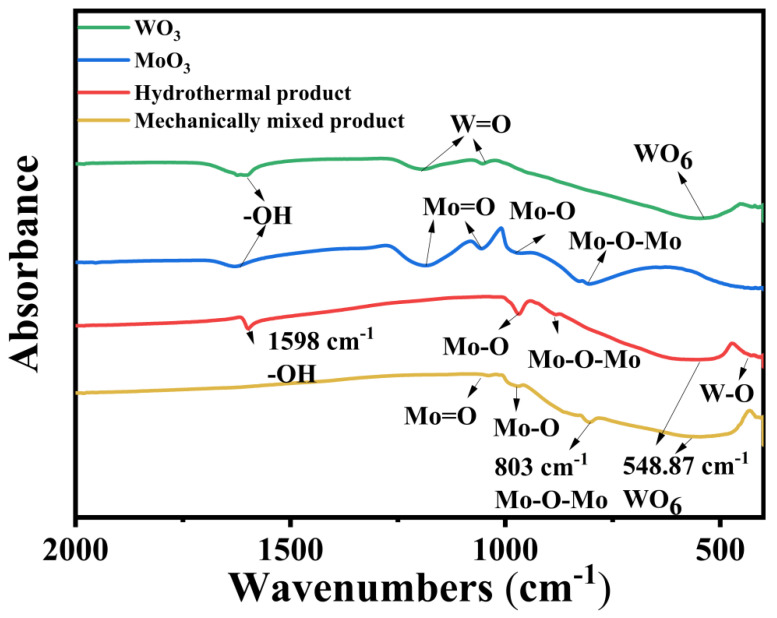
FT-IR spectra of WO_3_, MoO_3_ and MoO_3_/WO_3_ composites.

As shown in Figure 5, the similarity in the infrared spectral peak profiles between the hydrothermal product and the mechanical mixture, combined with the high separation factor observed for the mechanical mixture, provides strong evidence for the efficacy of tungsten–molybdenum separation in the hydrothermal product. Meanwhile, the FT-IR spectroscopy analysis provides conclusive evidence for the successful preparation of the Mo-W oxide composite material, consistent with the XRD results. Furthermore, this consistency may represent a key factor underlying the differences in separation efficiency observed between the hydrothermal product and the mechanical mixture. As illustrated in the spectrum of the final composite (n_MoO3_/n_WO3_ = 1:1), characteristic absorption bands corresponding to both molybdenum and tungsten oxides are present. The vibrations observed at 1038 cm^−1^, 969 cm^−1^, and 803 cm^−1^ are attributed to Mo=O stretching, Mo-O stretching, and O-Mo-O bridging vibrations, respectively. Notably, a distinct band at 548 cm^−1^, associated with the deformation vibration of WO_6_ octahedra, confirms the incorporation of tungsten species [18,19]. Crucially, a comparative analysis with the precursor materials (MoO_3_ and WO_3_) revealed significant peak shifts and the emergence of new features, indicating chemical interaction beyond simple physical mixing. For instance, the Mo=O stretching vibration, which appears at 1185 and 1055 cm^−1^ in the molybdenum precursor, shifts to 1038 cm^−1^ in the composite [20]. Similarly, the Mo-O stretch shifts from 961 cm^−1^ to 969 cm^−1^. The presence of a well-defined O-Mo-O bridge vibration at 803 cm^−1^ in the composite, which is also a key feature in the molybdenum precursor, further confirms the integration of the molybdenum oxide framework. The simultaneous presence of these Mo-related vibrations alongside the characteristic tungsten vibration (WO_6_ deformation at 548 cm^−1^) strongly demonstrates the successful co-existence of both metal oxides within a singular composite structure. The absence of intense, isolated -OH bending vibrations (seen at 1620 cm^−1^ in the precursors) in the composite spectrum also suggests successful processing and formation of the metal oxide network. In summary, the similarity in the infrared spectral peak profiles between the hydrothermal product and the mechanical mixture provides strong evidence for the effective separation of tungsten and molybdenum in the hydrothermal product. Moreover, the FT-IR results not only confirm the presence of both metal oxide components but also indicate the existence of a synergistic interaction between them. The successful synthesis of the Mo-W oxide composite may represent a key factor contributing to the differences in separation efficiency observed between the hydrothermal product and the mechanical mixture.

**Figure 6:**
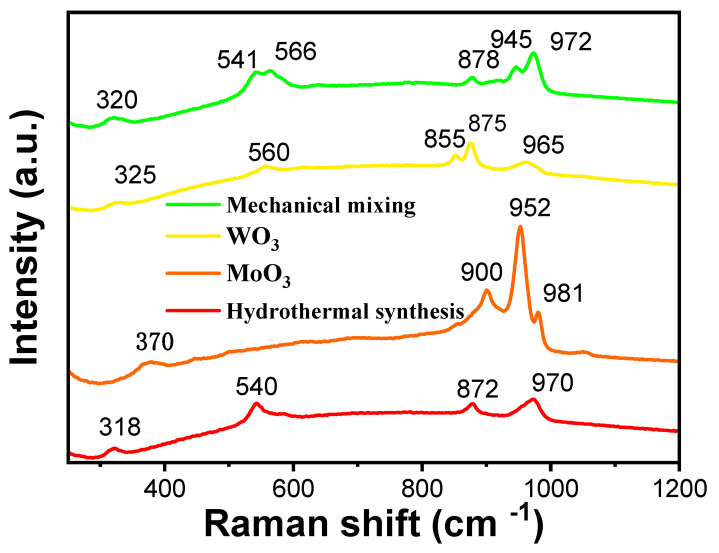
Raman spectra of WO_3_, MoO_3_, and their hydrothermal powder-synthesized and mechanically mixed composites from sodium molybdate and sodium tungstate at a Mo/W molar ratio of 1.

To investigate the peroxo species formed after the dissolution of Mo/W (100/1) mixed oxides in hydrogen peroxide, Raman analysis was performed on the filtrate obtained under conditions of n_H2O2_/n_Metal_ = 1, 80 °C, pH = 1.35 and 1 h leaching time, as shown in Figure 6. Pure WO_3_ exhibited characteristic Raman peaks at 855, 875, and 965 cm^−1^, assigned to W=O stretching vibrations of the WO_6_ octahedral units. Pure MoO_3_ showed intense peaks at 900, 952, and 981 cm^−1^, corresponding to Mo=O stretching vibrations of the MoO_6_ framework [21]. These peaks serve as structural fingerprints for distinguishing the two oxides. The Raman spectrum of machine mixing (n_WO3_/n_MoO3_ = 1:1) retained all characteristic peaks of pure WO_3_ and MoO_3_, with peak positions and intensities nearly identical to the pure phases. The WO_3_ peaks at 878/945 cm^−1^ and MoO_3_ peaks at 900/981 cm^−1^ were clearly resolved, with no significant shifts or broadening. This indicates that mechanical mixing only achieves physical dispersion of WO_3_ and MoO_3_, preserving their intrinsic crystalline structures and chemical bonds. The absence of interfacial interactions is critical for retaining the original coordination properties of each oxide—an essential prerequisite for separation via hydrogen peroxide (which relies on differential coordination with WO_3_/MoO_3_). In contrast, the hydrothermal method (n_WO3_/n_MoO3_ = 1:1) spectrum displays distinct deviations from the pure and mechanically mixed samples: (1) Enhanced MoO_3_ peaks: The MoO_3_ peak at 900 cm^−1^ (Mo=O stretching vibrations) increases in intensity compared to machine mixing, suggesting preferential growth or aggregation of MoO_3_ during hydrothermal treatment. (2) WO_3_ peak shift: The WO_3_ peak at 878 cm^−1^ (W-O-W bending vibration) shifts to 885 cm^−1^, accompanied by slight broadening. This shift is attributed to interface-induced strain from Mo-O-W bond formation, indicating chemical interactions between WO_3_ and MoO_3_ during deposition; (3) Synergistic modes: A new weak peak at 540 cm^−1^ is assigned to Mo-O-W bridge vibrations, providing direct evidence of heterointerface bonding [11,22]. Raman spectroscopy clearly differentiates the structural characteristics of MoO_3_/WO_3_ composites prepared by mechanical mixing and hydrothermal deposition. Mechanical mixing maintains the intrinsic structures of both oxides, making it compatible with tungsten/molybdenum separation via hydrogen peroxide. In contrast, hydrothermal deposition induces interfacial chemical interactions, which may alter the coordination environment of the oxides and reduce separation efficiency. These findings highlight the importance of structure-preserving preparation methods for retaining the functional properties required for selective separation [23].

**Figure 7:**
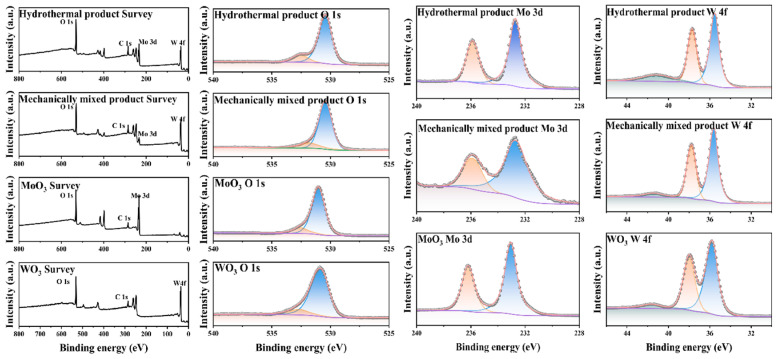
XPS spectra of WO_3_, MoO_3_, and their machine mixing (n_WO3_/n_MoO3_ = 1:1) and hydrothermally treated composites (n_WO3_/n_MoO3_ = 1:1).

To characterize the chemical states, interfacial interactions, and structural integrity of WO_3_, MoO_3_, and their composites (mechanically mixed (n_WO3_/n_MoO3_ = 1:1); hydrothermally (n_WO3_/n_MoO3_ = 1:1)), XPS was employed [24,25,26,27]. As shown in Figure 7, the W 4f spectrum exhibited a well-resolved doublet with W 4f_7/2_ at 35.6 eV, corresponding to the fully oxidized W^6+^ state (typical of WO_6_ octahedra). The O 1s peak at 530.2 eV was attributed to lattice oxygen (O^2−^), indicating a stoichiometric, defect-free structure. Meanwhile, the Mo 3d spectrum showed a Mo 3d_5/2_ peak at 232.5 eV, consistent with Mo^6+^ in MoO_6_ frameworks. The O 1s peak (530.2 eV) further confirmed lattice oxygen dominance. These peaks define the intrinsic chemical states of WO_3_ and MoO_3_, which are critical for their differential coordination with H_2_O_2_. For the mechanically mixed composite, the XPS spectra closely mirror those of pure WO_3_ and MoO_3_, with no significant binding energy shifts for W 4f or Mo 3d. These results indicate that mechanical mixing only achieves physical dispersion of WO_3_ and MoO_3_, preserving their intrinsic crystalline structures and chemical states. Therefore, the unaltered W^6+^/Mo^6+^ coordination environments retained their original affinity for H_2_O_2_, enabling selective separation via differential complexation. In contrast to the mechanically mixed sample, the hydrothermal method caused distinct deviations from the pure oxides, reflecting chemical interactions at the WO_3_/MoO_3_ interface. The Mo 3d_5/2_ peak shifted to 232.0 eV (ΔE = −0.5 eV) relative to pure MoO_3_, indicating partial reduction of Mo^6+^ to Mo^5+^. This is attributed to electron transfer in newly formed W-O-Mo bonds, a hallmark of interfacial coupling. The W 4f_7/2_ peak showed a slight downshift (0.3 eV) to 35.3 eV, suggesting trace reduction of W^6+^ to W^5+^ at the heterointerface. The O 1s spectrum revealed a higher proportion of defect oxygen (531.5 eV) and surface-adsorbed oxygen (533.5 eV) compared to machine mixing, reflecting increased structural defects caused by hydrothermal treatment. These changes alter the coordination environments of W and Mo: the reduced oxidation states (Mo^5+^/W^5+^) and defect sites modify the electronic structure of the oxides, potentially disrupting their original affinity for H_2_O_2_. This is detrimental to separation, as the process relies on the unique coordination properties of fully oxidized W^6+^/Mo^6+^. XPS analysis clearly differentiates the structural integrity of the two composites: (1) Mechanical mixing is a structure-preserving method that retains the intrinsic chemical states of WO_3_/MoO_3_, making it compatible with tungsten/molybdenum separation via H_2_O_2_. (2) Hydrothermal deposition induces interfacial interactions and chemical state changes, which may compromise separation selectivity by altering the oxides’ coordination environments. These findings highlight the importance of preparation method selection for maintaining the functional properties required for selective metal oxide separation.

In addition to the structural evidence obtained from Raman and XPS analyses, the pronounced difference in dissolution behavior between MoO_3_ and WO_3_ in hydrogen peroxide solution can also be interpreted from the perspective of coordination thermodynamics. Previous studies have shown that both Mo(VI) and W(VI) are capable of forming peroxo complexes in H_2_O_2_-containing systems; however, the thermodynamic stability of peroxomolybdate species is generally higher than that of the corresponding peroxotungstate species. Consequently, Mo(VI) exhibits a stronger coordination affinity toward H_2_O_2_ and readily forms stable peroxo complexes in solution, promoting its preferential dissolution and stabilization in the liquid phase. In contrast, the peroxo complexes of W(VI) are relatively less stable and are more prone to decomposition or reprecipitation, resulting in a significantly lower dissolution efficiency. Such differences in the thermodynamic stability of peroxide complexes have been reported previously and provide a reasonable explanation for the selective dissolution behavior observed in this study [11,28]. Therefore, the selective separation of Mo and W can be attributed to their different coordination tendencies and thermodynamic stability in hydrogen peroxide media.

**Figure 8:**
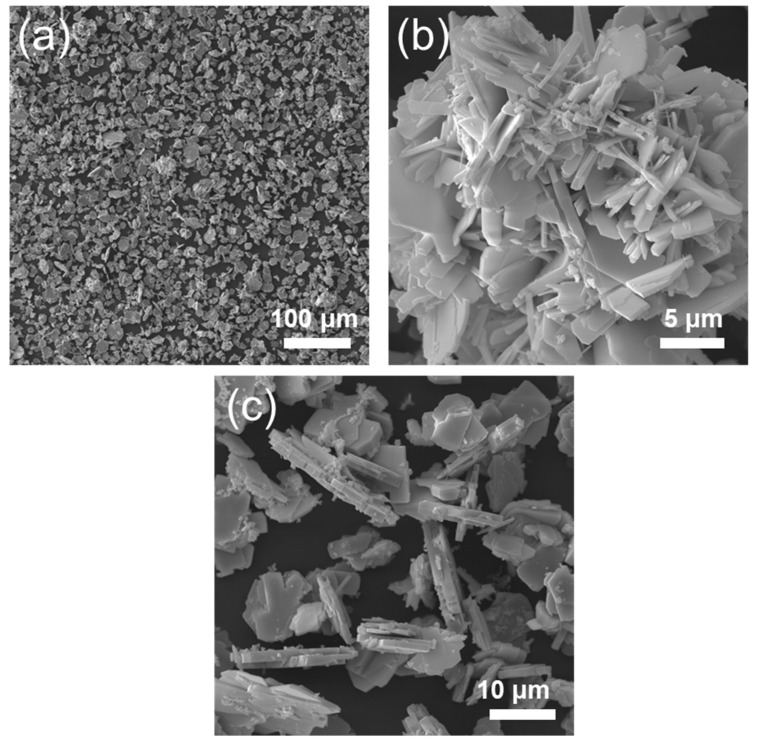
SEM images of hydrothermal powder synthesized from sodium molybdate and sodium tungstate at a Mo/W molar ratio of 1 (**a**–**c**).

SEM images (Figure 8a–c) revealed a hierarchical microstructure in which MoO_3_ particles (5–10 μm) were uniformly encapsulated by WO_3_ nanosheets (100–500 nm thick). The low-magnification image (Figure 8a) shows a dense, homogeneous distribution of the composite, while high-magnification views (Figure 8b) clarify a specific architecture: MoO_3_ cores are fully enwrapped by WO_3_ shells, with no exposed MoO_3_ surfaces detectable. This encapsulation is attributed to the preferential nucleation and growth of WO_3_ on MoO_3_ surfaces during synthesis (e.g., hydrothermal), driven by their surface energy differences.

### 3.3. Study on Separation Parameters and Their Optimization

#### 3.3.1. Effect of H_2_O_2_ Concentration

**Figure 9:**
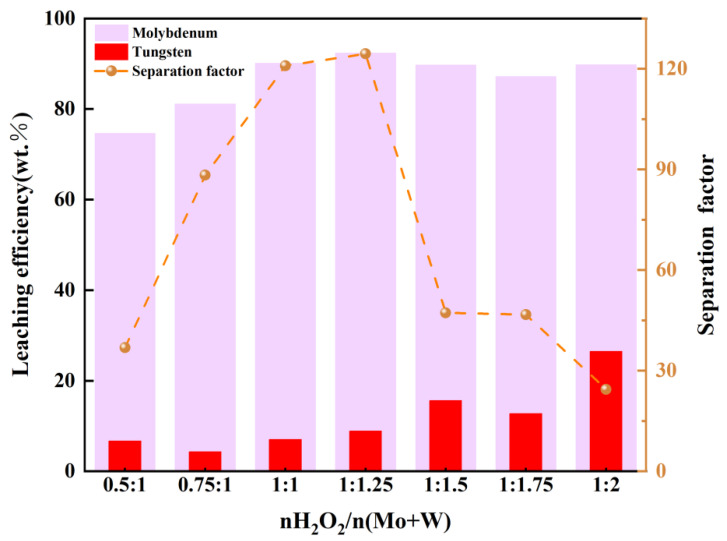
The influence of H_2_O_2_ dosage on leaching efficiency.

In each parallel experiment, 1 mL of the filtrate solution was introduced into 100 mL of the simulated solution, followed by periodic and precise collection of 10 mL samples for subsequent analysis. This study investigates the influence of H_2_O_2_ dosage on the W/Mo separation factor. Figure 9 illustrates the effect of H_2_O_2_ dosage, expressed as the molar ratio of H_2_O_2_ to total metals (nH_2_O_2_/n(Mo + W)), on the leaching efficiencies of tungsten (W) and molybdenum (Mo). The leaching efficiency of W increased gradually from 7% at nH_2_O_2_/n(Mo + W) = 0.5:1 to 27% at 1:2. A slight plateau (15–17%) observed between 1:1.5 and 1:1.75 suggests temporary saturation of W^6+^ coordination sites before further H_2_O_2_ addition promoted complexation. In contrast, Mo leaching reached a near-maximum value (92%) at nH_2_O_2_/n(Mo + W) = 1:1.25 and remained constant with additional oxidants, reflecting its strong affinity for H_2_O_2_, consistent with the reported higher stability of peroxo-Mo complexes ([Mo(O_2_)_4_]^2−^) compared to tungsten analogues [14]. The maximum separation factor (124) occurred at nH_2_O_2_/n(Mo + W) = 1:1.25, indicating optimal selectivity under these conditions. At this ratio, W leaching increased notably due to sufficient complexation, while Mo extraction had already plateaued at 92%, thereby maximizing the W/Mo ratio in the leachate. Further increasing the H_2_O_2_ dosage beyond 1:1.25 reduced the separation factor (to 24 at 1:2), as continued W leaching was offset by saturated Mo dissolution, diminishing relative selectivity. The higher affinity of Mo for H_2_O_2_ enables its rapid leaching at low dosages, whereas W requires greater oxidant concentrations to form soluble peroxo complexes. Thus, the molar ratio of 1:1.25 represents the optimum for achieving high W/Mo selectivity in the leachate.

#### 3.3.2. Effect of Reaction Time and Temperature

**Figure 10:**
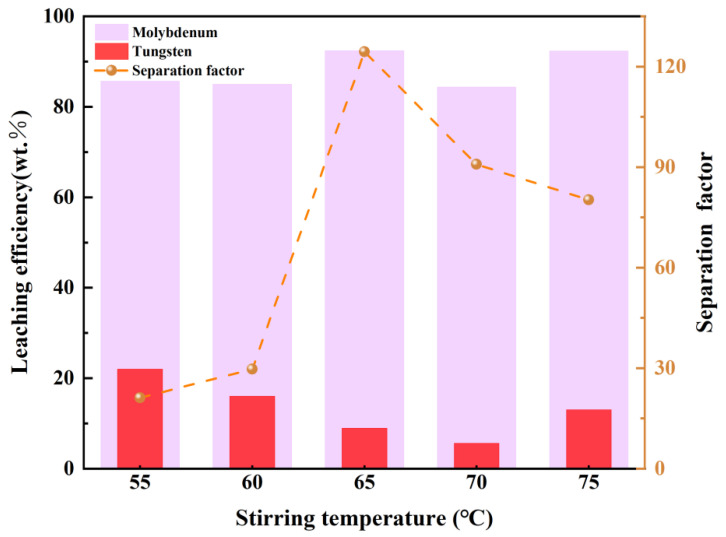
The influence of reaction temperature on separation efficiency.

In each parallel experiment of reaction time and temperature, 1 mL of the filtrate solution was introduced into 100 mL of the simulated solution, followed by periodic and precise collection of 10 mL samples for subsequent analysis. The influence of key process parameters, namely reaction temperature and duration, on Mo/W separation efficiency was systematically examined in the study. Figure 10 depicts the influence of stirring temperature (55–75 °C) on the leaching efficiencies of W and Mo, along with the corresponding separation factor—a key indicator of selective separation based on their differential coordination affinity with H_2_O_2_. The leaching efficiency of Mo remained stable (85%) between 55 and 60 °C, increased sharply to a maximum of 92% at 65 °C, and then fluctuated slightly (88–90%) at 70–75 °C. This behavior is attributed to the kinetics of peroxo complex formation ([Mo(O_2_)_4_]^2−^), which is enhanced by moderate temperature increases. At 55–60 °C, limited diffusion restricts Mo access to H_2_O_2_, despite its low decomposition rate. The optimum at 65 °C reflects improved diffusion and reaction kinetics. Beyond 65 °C, noticeable H_2_O_2_ decomposition (2H_2_O_2_ → 2H_2_O + O_2_) reduces effective oxidant concentration, slightly lowering Mo extraction. The partial recovery at 75 °C may result from intensified mixing partially counteracting H_2_O_2_ loss. In contrast, W leaching decreased monotonically from 22% at 55 °C to 5% at 70 °C, with a modest recovery to 13% at 75 °C. This decline stems from the inherently lower coordination affinity of W^6+^ toward H_2_O_2_ compared to Mo^6+^. Although limited complexation ([W(O_2_)_4_]^2−^) occurred at lower temperatures, rising temperatures accelerate H_2_O_2_ decomposition and emphasize the endothermic nature of tungstate peroxo formation, further suppressing leaching. The slight rebound at 75 °C is likely due to enhanced surface reactivity of W-containing phases, though oxide availability remains low. The separation factor reached a maximum of 85 at 65 °C, decreasing at both lower and higher temperatures. The results highlight the critical role of temperature in balancing peroxo complex formation kinetics and H_2_O_2_ stability. Mo^6+^, with its higher coordination affinity for H_2_O_2_, benefits from moderate temperature increments (65 °C) that enhance diffusion without excessive H_2_O_2_ decomposition. In contrast, W^6+^, with its lower affinity, is increasingly suppressed as temperature rises due to both H_2_O_2_ depletion and unfavorable thermodynamics. The optimal temperature of 65 °C thus represents a “sweet spot” where the differential coordination behavior of Mo/W is maximized, enabling efficient selective leaching [29].

**Figure 11:**
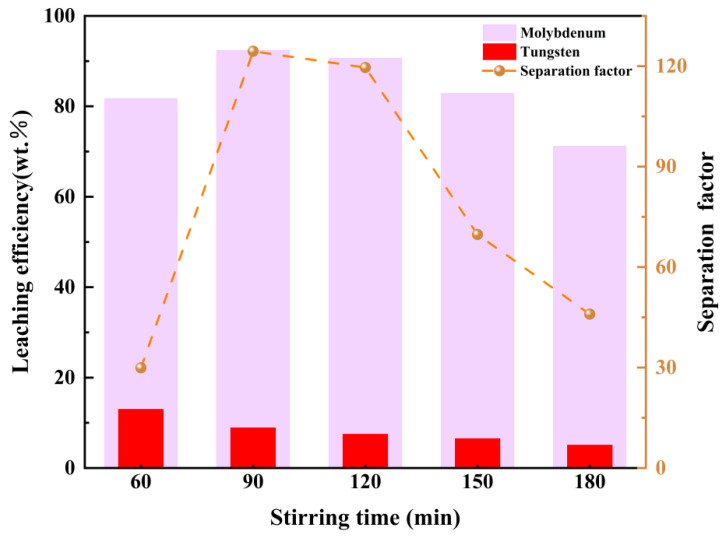
The influence of reaction time on separation efficiency.

In terms of reaction time, Figure 11 displays the effect of stirring time (60–180 min) on the leaching efficiencies of tungsten, molybdenum and the corresponding separation factor. The results elucidate the kinetic and thermodynamic interplay between Mo/W peroxo complex formation and separation efficiency, aligning with the premise of leveraging coordination differences for sustainable metal isolation. Mo leaching efficiency increases rapidly from 82% at 60 min to a maximum of 92% at 100 min, followed by a slight decline to 72% at 180 min. This trend is governed by the fast kinetics of Mo^6+^ peroxo complex formation ([Mo(O_2_)_4_]^2−^), which reaches thermodynamic equilibrium within 100 min due to the high coordination affinity of Mo^6+^ for H_2_O_2_. The subsequent decrease in Mo leaching is attributed to H_2_O_2_ thermal decomposition (2H_2_O_2_ → 2H_2_O + O_2_), which reduces the effective oxidant concentration over time. A minor contribution may also come from the depletion of surface-active Mo sites, limiting further complexation. Similarly, W leaching efficiency remains extremely low (<15%) throughout the experiment, decreasing monotonically from ~15% at 60 min to ~5% at 180 min. This persistent suppression is driven by two factors: (1) the lower coordination affinity of W^6+^ for H_2_O_2_ compared to Mo^6+^, which limits W^6+^ access to H_2_O_2_ in the presence of competing Mo^6+^; and (2) the instability of W peroxo complexes ([W(O_2_)_4_]^2−^), which are thermodynamically less stable than their Mo counterparts and prone to decomposition into insoluble WO_3_. As stirring time extends, these effects are exacerbated, resulting in minimal W leaching.

The separation factor reached a maximum of approximately 120 at 100 min, reflecting an optimal balance between efficient Mo complexation and sustained W suppression. This peak occurs when Mo leaching approaches saturation while W remains largely insoluble due to kinetic and thermodynamic barriers. Thus, 90 min represents the optimum stirring time, maximizing selectivity based on differential coordination behavior. These findings underscore the importance of time control in achieving efficient W/Mo separation via H_2_O_2_ leaching and provide a practical basis for scaling toward industrial applications where process efficiency and oxidant economy are critical.

#### 3.3.3. Effect of Mo/W Molar Ratio

**Figure 12:**
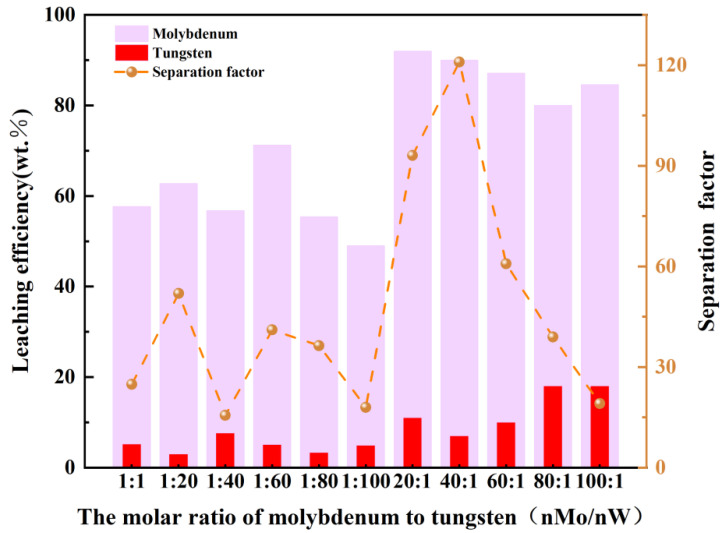
The influence of the molar ratio of Mo/W on separation efficiency.

Under these experimental conditions, Figure 12 shows the influence of the molar ratio of molybdenum to tungsten (nMo/nW, 1:1 to 100:1) on the leaching efficiencies of Mo and W, as well as the corresponding separation factor. The results elucidate how the Mo/W ratio modulates competitive peroxo complex formation and mass transfer, aligning with the premise of leveraging coordination differences for efficient metal isolation. Mo leaching efficiency increases non-linearly with increasing n_Mo_/n_W_, from ~60% at 1:1 to a maximum of ~95% at 20:1, followed by a gradual decline to ~85% at 100:1. This trend is governed by two key factors: (1) competitive coordination advantage: Mo^6+^ has a higher intrinsic coordination affinity for H_2_O_2_ than W^6+^, enabling preferential formation of stable [Mo(O_2_)_4_]^2−^ complexes as Mo concentration increases; (2) mass transfer limitations: at high n_Mo_/n_W_ (>40:1), excessive Mo loading leads to local H_2_O_2_ depletion and increased solution viscosity, reducing Mo^6+^ access to H_2_O_2_ and suppressing leaching. A minor dip in Mo leaching at 10:1 (~55%) is attributed to temporary H_2_O_2_ saturation, where Mo^6+^ coordination kinetics are hindered by limited oxidant availability. However, W leaching efficiency remains extremely low (<20%) throughout the experiment, decreasing slightly from ~10% at 1:1 to ~5% at 100:1. This persistent suppression is driven by W^6+^’s lower coordination affinity for H_2_O_2_ and competitive inhibition from Mo^6+^: as n_Mo_/n_W_ increases, Mo^6+^ dominates H_2_O_2_ coordination, leaving minimal oxidant for W^6+^ to form thermodynamically unstable [W(O_2_)_4_]^2−^ complexes. The negligible increase in W leaching at high n_Mo_/n_W_ (>80:1) (~18%) is likely due to marginal H_2_O_2_ availability after Mo saturation, with no meaningful contribution to W recovery. The results confirm the core premise of differential coordination: Mo^6+^’s higher affinity for H_2_O_2_ enables selective leaching, even at high Mo/W ratios. Key mechanistic takeaways include the following: (1) Competitive Coordination: Mo^6+^ outcompetes W^6+^ for H_2_O_2_, as evidenced by Mo leaching increasing with nMo/nW while W leaching remains suppressed. This is consistent with thermodynamic data showing that [Mo(O_2_)_4_]^2−^ has a higher formation constant than [W(O_2_)_4_]^2−^. (2) Ratio Optimization: The separation factor at 40:1 represents the optimal balance between Mo concentration (for efficient coordination) and H_2_O_2_ availability (to avoid depletion). At lower ratios (<20:1), Mo is insufficient to dominate H_2_O_2_ coordination; at higher ratios (>40:1), mass transfer limitations override coordination. (3) W Suppression: W’s persistent low leaching (<20%) confirms its weak interaction with H_2_O_2_, even in the presence of excess Mo. This is critical for maintaining separation selectivity, as W^6+^ cannot effectively compete for H_2_O_2_ under the studied conditions. This study provides a practical guideline for industrial Mo/W separation: optimizing the nMo/nW ratio to 40:1 achieves the highest separation factor (120) by maximizing Mo leaching and minimizing W co-leaching, reflecting the optimal balance between Mo’s coordination advantage and mass transfer efficiency and validating the premise of leveraging differential reactivity for sustainable metal isolation.

#### 3.3.4. Optimization

Based on a comprehensive synthesis of the separation effects influenced by the aforementioned factors, the optimal set of conditions was determined to be n_Mo_/n_W_ = 40, n_H2O2_/n_M_ = 1.25, a temperature of 65 °C, and a reaction time of 1.5 h, under which a separation factor of 124 was attained. Based on our mass balance calculations, we estimate the residual Mo in the W-enriched product to be approximately 76.94%. Direct solid-phase analysis will be included in future work to confirm these values. Compared to the maximum separation factor (112) achieved by the thermal decomposition method for separating peroxotungstic acid and peroxomolybdic acid [29], the advantage of our separation method is more pronounced.

**Figure 13:**
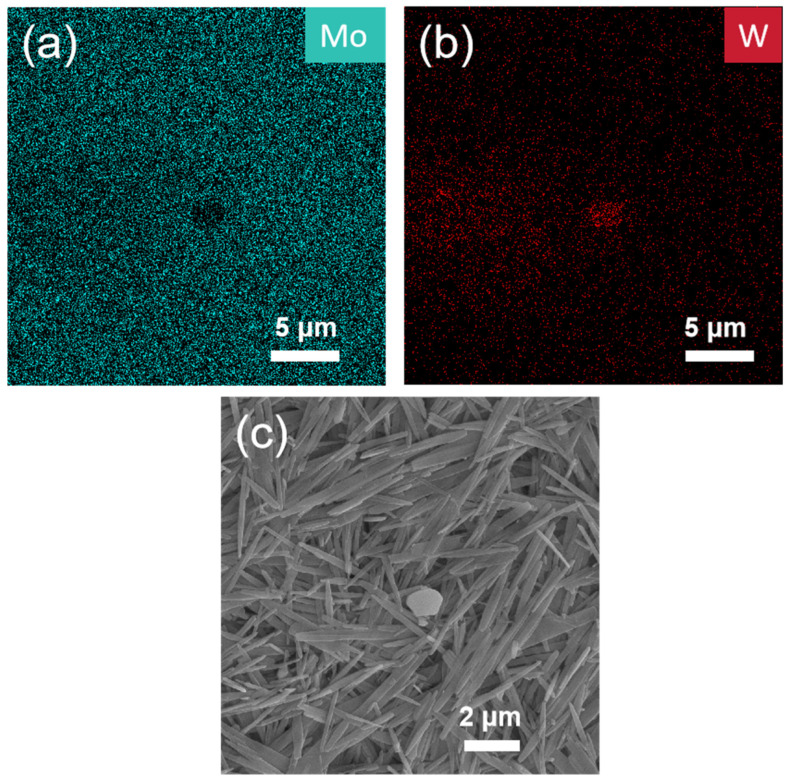
EDS and SEM images of hydrothermal powder synthesized from sodium molybdate and sodium tungstate at the optimal set of conditions (**a**–**c**).

Therefore, the filter residue obtained under these conditions was subjected to SEM and EDS analyses. Figure 13 reveals that, unlike the findings described in Section 3.2, both the molybdenum oxide (MoO_3_) and tungsten oxide (WO_3_) products generated under these conditions exhibited well-defined and discrete morphologies. This high degree of crystallinity and phase separation is likely a key factor contributing to the superior separation efficiency achieved under these parameters.

## 4. Conclusions

Based on the differential coordination behavior of tungsten and molybdenum with hydrogen peroxide, this study developed a novel approach for their selective separation from mixed oxides using H_2_O_2_ as a dissolving agent via mechanical mixing. Experimental results indicated that a separation factor as high as 2473 could be achieved with a pure MoO_3_/WO_3_ system prepared by mechanical mixing, confirming the significant difference in the coordination affinity of MoO_3_ and WO_3_ toward H_2_O_2_, which enables highly efficient tungsten–molybdenum separation. Furthermore, hydrothermal synthesis was employed to simulate the separation process under practical conditions, further validating the applicability of the proposed approach. By systematically evaluating the effects of multiple operational variables on separation performance, the optimal conditions were identified as n_Mo_/n_W_ = 40, n_H2O2_/n_M_ = 1.25, a temperature of 65 °C, and a reaction time of 1.5 h, under which a separation factor of 124 was attained. This process offers advantages such as low hydrogen peroxide consumption, a high separation factor, and cost-effectiveness, demonstrating promising potential for industrial application.

## Data Availability

The original contributions presented in this study are included in the article. Further inquiries can be directed to the corresponding author.
